# Stressors and Pain Over the Late-Life Course: Findings from Two Parent Longitudinal Studies of Aging and Health

**DOI:** 10.1093/geroni/igaa057.3383

**Published:** 2020-12-16

**Authors:** Penny Brennan

**Affiliations:** University of California, San Francisco, San Francisco, California, United States

## Abstract

With this study we sought to determine how older adults’ stressors influence their levels and rates of change in pain during the late-life span. We harmonized repeated measures data from two parent longitudinal studies of aging and health, Longitudinal Late-Life Health (LLLH; n=1,1884) and the Health and Retirement Study (HRS; n=7,703), to determine how participants’ stressor levels in the domains of finances, spouse, children, extended family, and friends, and in stressors overall, influenced their average levels and rates of change in painful conditions, pain severity, and pain interference over 13-year (LLLH) and 8-year (HRS) intervals. Participants’ within-person stressor levels declined somewhat, whereas their number of painful conditions, pain severity, pain interference, and prescription painkiller use increased steadily, over these intervals. In both the LLLH and HRS samples, participants who experienced higher average stressor levels over the 13- and 8-year intervals had more numerous painful conditions and higher pain severity over these intervals. In the HRS sample, they also experienced higher levels of pain interference. These effects occurred independent of the demographic characteristics of age, gender, and race. In general, participants’ stressor levels did not influence rates of increase in their pain. Gender and race had some moderating effects on associations between stressors and pain, but these occurred only within certain specific stressor and pain domains. These findings demonstrate an association between stressors and pain across the late-life course. Further research is needed to determine the mediating mechanisms that account for this association and the moderating factors that affect its strength.

